# Tonoplast proton pumps regulate nuclear spacing of female gametophytes *via* mediating polar auxin transport in *Arabidopsis*

**DOI:** 10.3389/fpls.2022.1006735

**Published:** 2022-09-13

**Authors:** Yu-Tong Jiang, Ji-Xuan Zheng, Rong-Han Li, Yu-Chen Wang, Jianxin Shi, Ali Ferjani, Wen-Hui Lin

**Affiliations:** ^1^Laboratory of Metabolic and Developmental Sciences, School of Life Sciences and Biotechnology, The Joint International Research, Shanghai Jiao Tong University, Shanghai, China; ^2^Zhiyuan College, Shanghai Jiao Tong University, Shanghai, China; ^3^Department of Biology, Tokyo Gakugei University, Koganei, Japan; ^4^Shanghai Collaborative Innovation Center of Agri-Seeds/Joint Center for Single Cell Biology, Shanghai Jiao Tong University, Shanghai, China

**Keywords:** plant vacuole, V-ATPase, female gametophyte, egg cell, central cell, endosperm

## Abstract

The vacuole is an important organelle with multiple functions in plants, and the tonoplast that wraps the vacuole also plays essential roles in intracellular trafficking and ion homeostasis. Previous studies found that tonoplast proton pumps regulate embryo development and morphogenesis through their effects on vacuole biogenesis and distribution, as well as polar auxin transport and concomitant auxin gradient. However, the precise roles of the tonoplast proton pumps in gametophyte development remain unclear. Here we demonstrated that the lack of two types of tonoplast proton pumps or the absence of V-ATPase alone leads to abnormal development and nuclear localization of female gametophyte (FG), and slowed endosperm nuclei division after fertilization of the central cell. We further revealed that V-ATPase regulates auxin levels in ovules through coordinating the content and localization of PIN-FORMED 1 (PIN1) protein, hence influencing nuclear spacing between centra cell and egg cell, and subsequent endosperm development. Collectively, our findings revealed a crucial role of V-ATPase in auxin-mediated FG development in *Arabidopsis* and expanded our understanding of the functions of tonoplast proton pumps in seed plants reproductive development.

## Introduction

The vacuole, as an organelle with a single membrane, forms compartments and is a sophisticated endomembrane system unique to plant cells (Marty, 1999). As an important barrier for material exchange into and out of the vacuole, the vacuolar membrane, also referred to as tonoplast, harbors a rich variety of transporters, among which the tonoplast proton pumps are the most fundamental proteins. There are two distinct proton pumps, namely the vacuolar H^+^-pyrophosphatase (V-PPase, EC 3.6.1.1) and the vacuolar H^+^-ATPase (V-ATPase, EC 3.6.4.10) (Hedrich et al., 1989), that have been reported in the literature, both exhibit completely different structural and functional properties. The V-PPase and V-ATPase catalyze the hydrolysis of Mg^2+^-PPi and Mg^2+^-ATP complex, respectively, and extract energy for pumping H^+^ ions into the vacuolar lumen, thus maintaining a proton gradient (ΔpH) across the tonoplast (Maeshima, 2000; Martinoia et al., 2007).

Whereas V-PPase has a relatively simple structure and is encoded by *AVP1/FUGU5* in *Arabidopsis thaliana* (*Arabidopsis* hereafter) (Ferjani et al., 2011; Kriegel et al., 2015; Jiang et al., 2020), the V-ATPase is a versatile protein complex that broadly occurs in all cell types (Marshansky and Futai, 2008). It exhibits a unique differential targeting on endomembrane compartments, which is mediated by different isoforms of its subunit a (Lupanga et al., 2020). In *Arabidopsis*, V-ATPase complex containing VHA-a2 or VHA-a3 is localized at the tonoplast (Krebs et al., 2010; Jiang et al., 2020). According to previous studies, *fugu5-1*, a point mutation line of AVP1, lacks V-PPase activity and exhibits basically the same growth conditions as wild type (Kriegel et al., 2015; Jiang et al., 2020); *vha2* (*vha-a2 vha-a3*), T-DNA insertion line of VHA-a2 and VHA-a3, lacks V-ATPase activity at the tonoplast and exhibits overall impaired growth phenotypes (Krebs et al., 2010); and *fap3*, the triple mutant obtained by crossing *fugu5-1* with *vha2*, lacks both V-PPase and V-ATPase functions, and displays a hindered vegetative growth and an aberrant cotyledon boundary during embryogenesis due to blocked PIN-FORMED (PIN)1-mediated auxin transport (Kriegel et al., 2015; Jiang et al., 2020). Tonoplast proton pumps regulate embryo development through PIN1-mediated auxin transportation. However, tracking back along the reproductive developmental process, gametophyte development of plants lacking both tonoplast proton pumps remained poorly understood.

During the reproductive growth of angiosperms, male gametophytes are always produced in great redundancy, while female gametophytes (FGs) are produced in less abundance due to evolutionary specialization. Thus, FG development is indispensable for the generational alternation in higher plants. In most angiosperms with polygonum-type FGs, megaspore mother cell (MMC) undergoes meiosis to produce four megaspores and one of them becomes the functional megaspore, which gives rise to an FG. The development process of FGs can be divided into 8 stages from FG1 to FG8 (Christensen et al., 1997). At FG6 stage, with the fusion of polar nuclei, a fixed pattern of seven cells is formed: an egg cell and two synergid cells at the micropylar end, a central cell in the central area, and three antipodal cells at the chalazal end (Yang et al., 2010). The development of FGs determines the portion of fertile embryos and the number of seeds produced. Besides, FGs also influence many aspects of plant reproduction, including male-female crosstalk and maternal effects on seed development, highlighting the significance of untangling its developmental regulatory network.

Plant hormones, such as brassinosteroids (BRs), gibberellin (GA), cytokinin (CK), and auxin have been shown to play essential roles in FGs development (Pérez-España et al., 2011; Yuan et al., 2016; Gomez et al., 2020). BR, CK, and GA generally contribute indirectly to FG development through their effects on specific proteins that manipulate auxin levels among embryo sacs and change the FG fate (Pagnussat et al., 2009; Luca et al., 2013; Panoli et al., 2015). Auxin, due to its ubiquitous distribution and long-range mobility, has a crucial direct impact on FG development; as a signal molecule, auxin synthesis, transport, and dynamic concentration gradient are deeply involved in the whole process of female reproductive growth (Serbes et al., 2019). It is reported that auxin also acts as a positional cue for cell fate determination, and that the accurate positioning of FG nuclei is important for their identity (Sun et al., 2021). The build-up of auxin gradient relies on polar auxin transportation (PAT) through various organelles and the endomembrane transport system. Previously, we showed that tonoplast proton pumps regulate the intracellular trafficking of PIN1 protein, hence influencing auxin homeostasis during embryonic development in *Arabidopsis* (Jiang et al., 2020). Whether tonoplast proton pumps functions in FGs development, however, remains to be explored.

In this work, to fill knowledge gap about the precise function of tonoplast proton pumps in *Arabidopsis* FGs development, we conducted a multiscale phenotypic analysis of mutants lacking both V-PPase and V-ATPase activity, and found that these mutants exhibit an abnormal nuclear-spacing between FG nucleus and delayed endosperm division. We are also proposing a working model linking tonoplast proton pumps and PAT in FGs developmental process. Our results provide novel insights into roles of tonoplast proton pumps in the reproductive developmental process.

## Materials and methods

### Plant material and growth conditions

All materials used in this study were generated in *Arabidopsis* ecotype Columbia 0 (Col-0) background. Seeds were soaked with sterile water for 1 min, surface sterilized with a 3% NaClO solution for 5 min, then rinsed 5 times in sterile distilled water. Sterile seeds were grown on agar plates containing 1/2 Murashige and Skoog (MS) medium [2.215 g L^–1^, 0.75% agar (w/v), 1.5% sucrose (w/v), pH = 5.75]. Plates were incubated in the dark at 4°C for 2 days and cultured in a growth chamber with a photoperiod of 16 h/8 h, light/dark cycling, and a temperature of 22 ± 1°C. After 7 days, the plants on the dishes were transferred to a mixture of soil:vermiculite:perlite (10:10:1). In this paper, to make the mutant *vha2* grow better during vegetative stages, both *vha2* and its contemporaneous control plants were grown under 24-h long-day light.

### Identification of the T-DNA insertion lines and construction of transgenic plants

For mutants of tonoplast proton pumps used in this study, *vha2* [crossing *vha-a2* (SALK_142642) with *vha-a3* (SALK_029786) from Salk Institute for Biological Studies] and *fap3* [obtained by crossing *fugu5-1* with *vha2*] were both described in detail in our previous work, as well as *ProPIN1:PIN1-YFP* and R2D2 marker line (Jiang et al., 2020; Yu et al., 2020). For quantification of fluorescent signals of R2D2, we used the DII-Venus relative to mDII-ntdTomato for the semi-quantitative analysis of auxin level in plants, which is represented by DII/mDII (Benková et al., 2003; Liao et al., 2015). The construction of *ProES1:H2B-GFP* were based on the previously published literature (Pagnussat et al., 2007). To generate the *ProVHA-a3:VHA-a3-GFP*, the promoter and genomic DNA sequences were amplified by PCR using genomic DNA of Col as templates, and then cloned into the pHB vectors (Biovector NTCC Inc.) digested by *Eco*RI and *Hin*dIII, respectively. The sequences of oligonucleotide primer sets used are listed in Supplementary Table 1.

### RNA extraction and qRT-PCR assays

The materials used for total RNA extraction in this study were inflorescence apices of *Arabidopsis*, which were harvested with tweezers and quick-frozen in liquid nitrogen. Subsequent RNA extraction and reverse transcription were performed using the RNAprep Pure Plant Kit (TIANGEN) and ReverAid First Strand cDNA Synthesis Kit (Thermo Fisher Scientific) following the manufacturers’ protocols. qRT-PCR assays were conducted for three technical replicates of three biological replicates, and the results are shown as the mean ± standard deviations. The details were carried out as described previously (Zhang et al., 2016). The sequences of oligonucleotide primer sets used are listed in Supplementary Table 1.

### *In vitro* treatment assays

To analyze the development status of FG in different stages upon 1-naphthylacetic acid (NAA; Sigma-Aldrich 317918) and picloram (Sigma-Aldrich 1918021), vigorously growing inflorescence apices were used as experimental materials. Floral buds larger than stage 10 were removed and the remaining inflorescences apices were immersed in 0.01% (v/v) Silwet L-77 solutions containing 1 μM NAA, or 1 μM picloram. After 24 h, wash the inflorescences apices with distilled water to remove residual agent. The materials were collected after 2 days.

### Confocal microscopy

Confocal laser-scanning microscopy (CLSM) observation of male and FG was strictly performed as described previously (Christensen et al., 1997), but with the modification that we used the Upright Laser Confocal Microscope (Nikon & Nikon Ni-E A1 HD25). For the collection of male and FGs, we took the vigorously growing buds from outside to inside of inflorescence apices. For the analysis of flowers containing GFP constructs, we first carefully peeled the pistils from the flowers in different periods and fixed them in 4% paraformaldehyde for 30 min and then immersed in Clearsee agent according to Clearsee method (Kurihara et al., 2015). Cell walls were stained with 0.02% Fluorescent Brightener 28 (FB28) in Clearsee agent for over 3 h, as described in our previous study (Zu et al., 2022). Before observation, materials were soaked in Clearsee reagent to wash away the cell wall stain for more than half an hour. For the ovule wrapped inside the pistils, we tapped the corresponding position of the coverslip with the tip of the tweezers to exposed the ovule. For CLSM, GFP was excited with an argon laser at a wavelength of 488 nm, and emission was detected between 500 and 530 nm. FB28 was excited with an argon laser at a wavelength of 395 nm, and emission was detected between 450 and 480 nm. Measurements of nuclear spacing and fluorescence intensity were obtained using the Image J software.

### Male gametophyte observation assays

Male gametophyte analyses, including Alexander staining, scanning electron microscopy (SEM), *in vitro* culture of pollen grains, DAPI staining of pollen grains and aniline blue staining of pollen tube were performed as described previously (Johnson-Brousseau and McCormick, 2004; Li et al., 2013).

### Quantification and statistical analysis

Data analysis and statistical graphs were carried out using GraphPad Prism 8 and Microsoft Office Excel software. For comparison between two groups, Two-tailed Student’s *t*-test was used, and for more than two groups, Duncan’s test was used. The specific statistical methods for each assay are described in figure legends. Quantification of the phenotypes were performed using the ImageJ software.^1^

### Accession numbers

*Arabidopsis* genes mentioned in this article are as follows: *AVP1/FUGU5*, AT1G15690; *VHA-a2*, AT2G21410; *VHA-a3*, AT4G39080; *PIN1*, AT1G73590; *PIN3*, AT1G70940; *PIN4*, AT2G01420; *ARF1*, AT1G59750; *ARF2*, AT5G62000; *ARF3*, AT2G33860; *ARF4*, AT5G60450; *ARF5*, AT1G19850; *YUC2*, AT4G13260; *YUC5*, AT5G43890; *YUC8*, AT4G28720; *YUC9*, AT1G04180.

## Results

### Vacuolar H^+^-ATPase deficiency results in ovule and seed abortion

To explore the role of the tonoplast proton pumps in gametophyte development of *Arabidopsis*, we focused on the reproductive development of *fap3* mutant lacking both V-PPase and V-ATPase activities. Most of mature flowers at stage 13 (72%, 112/156) obviously had morphological abnormalities (Supplementary Figure 1A) and 97% (118/121) ovules of *fap3* were aborted before fertilization (Supplementary Figure 1B). Further examinations with Alexander staining, CLSM, and SEM on pollen grains revealed that 72% (64/89) pollen grains of *fap3* are not fertile due to defective pollen development (Supplementary Figures 1C–E), indicating that loss-of-function of both V-PPase and V-ATPase activities severely affects male gametophyte development as well. Based on CLSM observation and statistics, only 2% (3/121) FGs of *fap3* could develop normally, and the rest prematurely aborted (Supplementary Figure 1F), confirming the roles of both V-PPase and V-ATPase in FG development. Collectively, the above results indicated that the lack of two tonoplast proton pumps can have extremely serious consequences for the entire process of plant reproduction. Therefore, *fap3* is unlikely an ideal material for studying the role of tonoplast proton pumps in plant FG development. Since the overall plant growth, male and female transmission of *fugu5-1* are comparable to those of the wild type Col-0 (Jiang et al., 2020; Supplementary Table 1), we next focused our study on V-ATPase mutant *vha2*.

**FIGURE 1:**
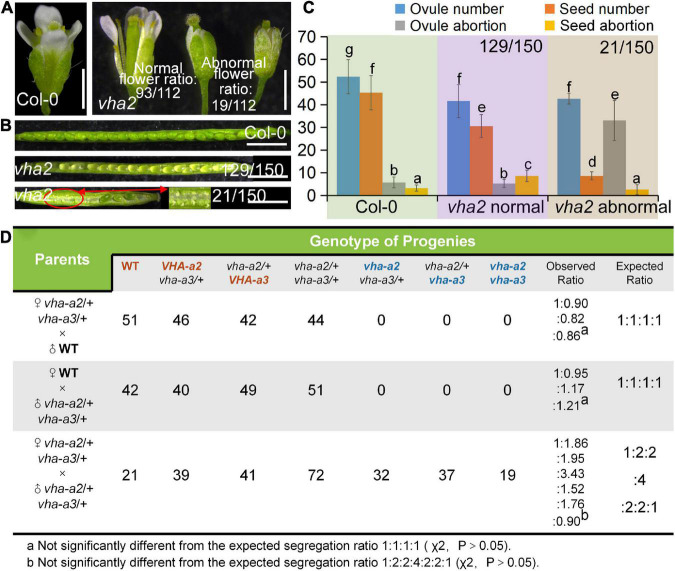
Lack of V-ATPase results in abnormal embryonic development. **(A)** Flower development at stage-13 in Col-0 and *vha2.* Statistics on the ratio of normal and abnormal microspores in *vha2* are shown in each image. Bar = 1 mm. **(B)** Seed development in Col-0 and *vha2.* Two types of siliques of *vha2* correspond to those in **(A)**. **(C)** Statistics analysis of ovule number, seed number, ovule abortion, and seed abortion in Col-0 and *vha2.* Bars represent the mean ± SD of three biological replicates (*n* = 30). Lowercase letters indicate statistically significant differences between different stages (*P*< 0.05). Theoretically, ovule number = seed number + ovule abortion + seed abortion. **(D)** Both male and female transmission of *vha-a2*, and *vha-a3* are comparable to *VHA-a2* and *VHA-a3* of WT. Genotypes consistent with wild type are marked in red font, homozygous mutant genotypes are marked in blue font, and heterozygous mutant genotypes are marked in black font.

As mentioned above, while VHA-a1 mediates TGN/EE targeting of V-ATPase, VHA-a2 and VHA-a3 mediate V-ATPase’s tonoplastic localization (Krebs et al., 2010; Kriegel et al., 2015; Luo et al., 2015; Lupanga et al., 2020). Therefore, *vha2* was used to investigate its specific role in plant gametophyte development. Considering that an extended illumination time can improve vegetative growth of *vha2* (Krebs et al., 2010), *vha2* mutants were grown under continuous light conditions, which greatly improved inflorescence growth to a level almost indistinguishable from Col-0 (Supplementary Figure 2) and eased our analyses. Interestingly, most flowers of *vha2* displayed normal morphologies, as only 17% (19/112) of them were abnormal (Figure 1A). Correspondingly, most siliques elongated normally and bore seeds, and only 14% (21/150) of them were obviously shorter (Figure 1B and Supplementary Figure 2). Consistently, *vha2* abnormal siliques had a higher ovule abortion rate and developed only few seeds (Figures 1B,C). As the stigma was morphologically defective (Supplementary Figure 3A), when abnormal *vha2* pistils were hand-pollinated with the Col-0 pollen, ovules could develop till the FG7 stage and remained unfertilized as examined at 48 h after pollination (HAP) (Supplementary Figure 3B). Hence, it appears that the premature abortion prior to fertilization could be derived from abnormal growth of the pollen tube on the defective stigma (Supplementary Figure 3C). Based on the above observations, morphologically normal *vha2* flowers were mainly used in our following experiments.

**FIGURE 2:**
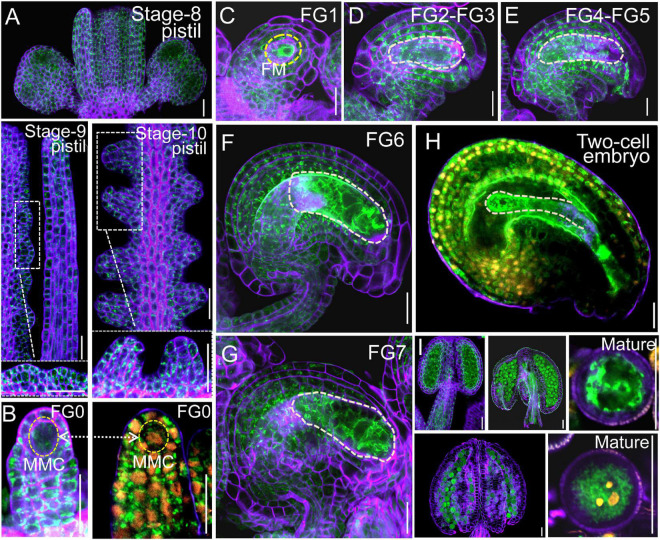
*VHA-a3* is constitutively expressed in reproductive organs. Representative stage-8, stage-9, stage-10 pistil **(A)**, ovules during FG0 stage **(B)**, FG1 stage **(C)**, FG2-FG3 stage **(D)**, FG4-FG5 stage **(E)**, FG6 stage **(F)**, FG7 stage **(G)**, two-cell embryo **(H)**, anthers and mature pollen grain **(I)** from the *Pro VHA-a3: VHA-a3-GFP* plants. The nuclear staining with DAPI in FG0 stage **(B)** to demonstrate that MMC is present in this developmental stage. Yellow dotted circles in **(D–G)** indicate the embryo sac and in **(H)** indicate the two-cell embryo; Orange dots indicate the nucleus by DAPI staining of mature pollen grain. The purple part is the outline of pollen grains stained with FM4-64. Bars = 20 μm. MMC megaspore mother cell, FM functional megaspore.

Next, we examined the male and female transmission of *vha-a2* and *vha-a3* and found that transmission of the *vha-a2* and *vha-a3* mutant alleles is comparable to *VHA-A2* and *VHA-A3* in WT alleles (Figure 1D). For a more rigorous follow-up study, we need to demonstrate that *VHA-a2* and *VHA-a3* are expressed during gametophyte development. So we examined V-ATPase expression by constructing a transgenic line for *VHA-a3*, the predominant form of the a subunit on the tonoplast, which is fully redundant with VHA-a2. Fluorescence images of a *ProVHA-a3: VHA-a3-GFP* transgenic line confirmed the expression of V-ATPase during the whole process of *Arabidopsis* gametophyte development and early embryo development as well (Figures 2A–I).

### Loss of vacuolar H^+^-ATPase activity affects pollen development

**FIGURE 3:**
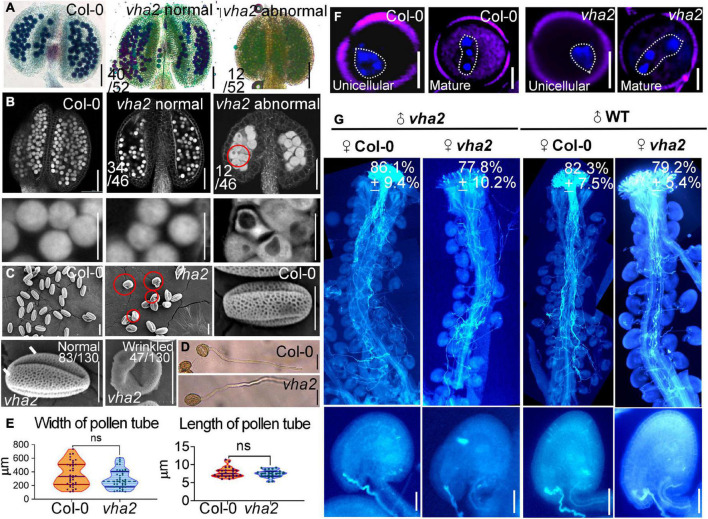
Lack of V-ATPase affects pollen development. **(A)** Alexander’s staining of pollen from Col-0 and *vha2.* The purplish red color indicates viable pollen. Bar = 100 μm. **(B)** Anthers visualized by CLSM. Col-0 and *vha2* anthers at stage 12. Statistics on the ratio of normal and abnormal flowers in *vha2* are shown in each image. Scale bars represent 100 μm (main image) and 10 μm (partial enlargement). Red circle indicates the pollen grain that should have matured was stagnant at PMI stage with big vacuole. **(C)** SEMs observation on pollen grains from Col-0 and *vha2.* The scale bar is marked in each image. Bars = 10 μm. **(D)** A tetrad of mature pollen of Col-0 and *vha2* from in vitro pollen germination. Bars = 50 μm. **(E)** Quantitative analysis of pollen germination. Results shown are means ± standard errors (SE, *n* = 3). In total, 30 tetrads were examined for each genotype. **(F)** DAPI staining of unicellular and mature pollen grains from Col and *vha2.* The purple part is the outline of pollen grains stained with FM4-64. Bars = 5 μm. **(G)** Aniline blue staining of Col-0 and *vha2* pistils at 24 h after pollination (HAP) with Col-0 and *vha2* pollen. The whole pistil was overlaid by three overlapping images with Photoshop (Adobe). Numbers in **(G)** are quantification of targeted ovules out of total ovules. Results are means ± SD (*n* = 15). Bars = 20 μm.

*vha2* normal siliques wrapped normal ovules, but the ratio of seed abortion in *vha2* normal siliques was higher than that of Col-0 (Figures 1B,C). To gain insights into the seed abortion in *vha2* normal siliques, we further examined male and FG development in *vha2*. For male fertility, *in vitro* pollen germination combined with alexander staining, fluorescence microscopy and SEM were performed to examine the pollen viability. 77% (40/52) anthers from *vha2* were stained purplish red by Alexander dye that was consistent with Col-0 (Figure 3A), while only 23% (12/52) anthers showed light blue pollen coloration (Figure 3A), indicating that most pollen grains were vigorous. Further CLSM implied that pollen grains with large vacuoles in infertile anthers are arrested in pollen mitosis I (PMI) stage (Figure 3B). SEM images of *vha2* revealed that the majority of pollen grains (64%, 83/130) are oval-shaped similar to Col-0 (Figure 3C), and that the remaining portion of pollen grains are slightly wrinkled (Figure 3C). *In vitro* pollen germination demonstrated that there are no significant differences between *vha2* and Col-0 regarding pollen tube length and width (Figures 3D,E). Therefore, the slightly wrinkled pollens had similar fertility to Col-0 normal ones. Furthermore, DAPI staining further demonstrated that *vha2* mature pollen has a normal nuclear division (Figure 3F). The abovementioned results implied that even though a small portion of pollens are slightly wrinkled, the loss of V-ATPase function does not significantly affect pollen fertility.

When Col-0 and *vha2* pistils were pollinated with Col-0 and *vha2* pollen separately, most ovules in Col-0 and *vha2* pistils were targeted by the pollen tubes examined at 24 HAP (Figure 3G). This result indicated that *vha2* has a comparable pollen tube guidance and reception to that of the Col-0, therefore, we focused our study on the development of FGs to further explore the mechanism behind seed abortion in normal *vha2* siliques.

### Vacuolar H^+^-ATPase deficiency leads to aberrant nuclear spacing during female gametophyte development and affects endosperm nuclei division rate

**FIGURE 4:**
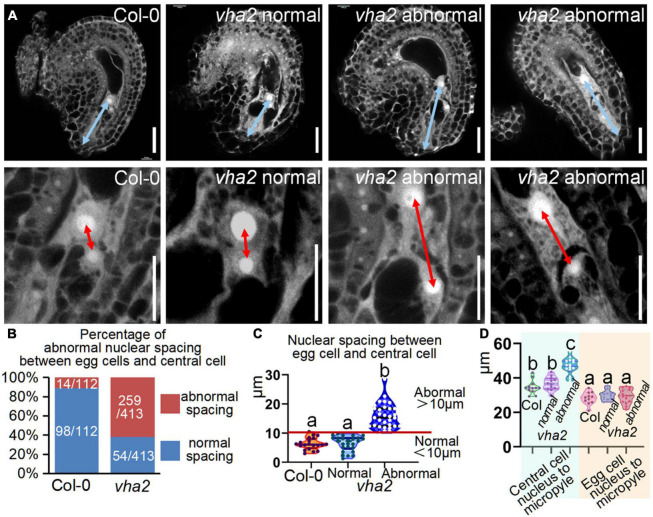
Lack of V-ATPase enlarges nuclear spacing of egg cells and central cells. **(A)** Embryo sac development of Col-0 and *vha2* at late-FG6 stage visualized by fluorescence microscopy. The picture below is an enlarged view of the nuclear spacing between the egg cell and central cell in the above image. The blue double arrows represent the distance from the central cell nucleus to the micropylar end, and the red double arrows in enlarged views represent nuclear spacing between egg cell and central cell. Bars = 20 μm. **(B)** Statistics analysis of the proportion of abnormal nuclear spacing between egg cell and central cell in Col-0 and *vha2.* The number of ovules counted and the actual ratio is marked in the image. **(C)** Statistics analysis of nuclear spacing between egg cell and central cell in Col-0 and *vha2*. Below the red line is defined as normal spacing if the nuclear spacing between egg cell and central cell is less than 10 μm, and it was considered abnormal spacing if spacing exceeds 10 μm. Lowercase letters indicate statistically significant differences between different stages (*P*< 0.05). 100 ovules shown in the statistical graph come from 20 different inflorescence apices. **(D)** Statistics analysis of the distance from the central cell nucleus or egg cell nucleus to the micropylar end in Col and *vha2.* Lowercase letters indicate statistically significant differences between different stages (*P*< 0.05). 30 ovules shown in the statistical graph come from 3 different inflorescence apices.

To see if there are defects in *vha2* FGs, we examined *vha2* ovules at different developmental stages using CLSM. There was no detectable morphological difference between WT and *vha2* FGs until FG6 stage (Supplementary Figure 4 and Figure 4A). During the transition of late FG6 to early FG7, the distance between central cell and egg cell nuclei in more than half of *vha2* embryo sacs was remarkably larger than that of Col-0 (Figures 4A–C). In *Arabidopsis*, late FG6-to-FG7 transition is an important step for embryo sac cell-fate decision and fertilization preparation. During this period, the pattern of eight nuclei and seven cells emerges, antipodal cells at chalaza end degenerates, and the distance between the central cell and egg cell nuclei is further shortened, preparing FGs for fertilization (Christensen et al., 1997; Sundaresan and Alandete-Saez, 2010).

Based on statistics of the distance in Col-0, we arbitrarily designated a distance less than 10 μm as normal spacing, and a distance larger than that as abnormal spacing, for convenient comparison and description. Under this criterion, the proportion of abnormal spacing in *vha2* was more than a half, while that in the wild type was about 10% (Figures 4B,C). Nuclear spacing abnormity was clearly associated with the failed movement of the central cell to the proper position at the micropyle end (Figure 4D). This increased nuclear spacing phenotype was also observed in abnormal embryo sacs in *fap3* (Supplementary Figure 5). Considering that *vha2* exhibited higher premature seed abortion rate compared with Col-0 (Figure 1C) and that the fusion of sperm and diploid central cell during double fertilization is essential for subsequent embryo, endosperm, and even seedling development (Portereiko et al., 2006; Wu et al., 2012), it is reasonable to assume that the distance between the nuclei of central cell and the egg cell that is affected by V-ATPase, may influence the fertilization process as well as the following division of endosperm nuclei.

**FIGURE 5:**
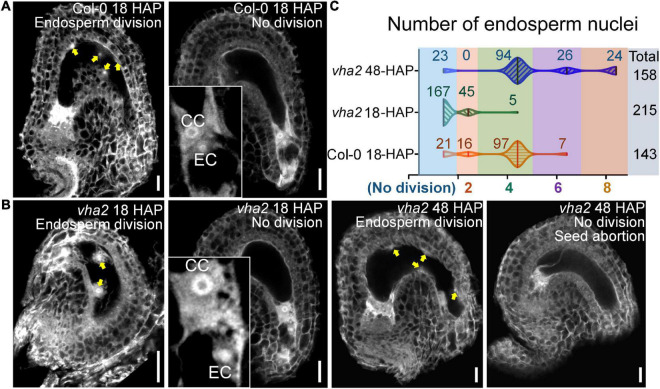
Lack of V-ATPase affects the division rate of endosperm nuclei. **(A)** Endosperm nuclei of Col-0 seeds at 18 h after pollination (HAP). **(B)** Endosperm nuclei of *vha2* seeds at 18 HAP and 48 HAP. Yellow arrows in **(A,B)** indicate the endosperm nuclei. EC egg cell, CC central cell, graphs represent typical phenotype of all samples. Bars = 20 μm. **(C)** Quantitative analysis of endosperm nuclei number in Col-0 and *vha2.* Seeds shown in the statistical graph come from 5 different inflorescence apices.

To formally test this hypothesis, we compared the endosperm development of *vha2* to that of Col-0 at 18 HAP using CLSM. At 18 HAP, most egg cells and central cells were already fertilized, and endosperms consisted of four to eight nuclei in Col-0 (Figures 5A,C). In contrast, at the same stage, ∼80% egg cells and central cells remained unfertilized and ∼20% seeds had two endosperm nuclei in *vha2.* Until at 48 HAP, most *vha2* endosperms contained four to eight nuclei (Figures 5B,C), while around 15% (23/158) nuclei of *vha2* endosperms did not divide, which was in line with the slightly increased ratio of seed abortion in *vha2* normal siliques (Figure 1C).

The above results indicated that V-ATPase deficiency leads to the increased nuclear spacing between the central and the egg cells in mature FGs, thus slowing down subsequent division of endosperm nuclei. These findings also demonstrated that nuclear spacing in mature FGs is essential for subsequent fertilization and seed development. Given that auxin acts as a positional cue for individual cell specification during FG6-to-FG7 stages (Sun et al., 2021), and that V-ATPase is a modulator of the polar transport and distribution of auxin in *Arabidopsis* embryos and seedlings (Jiang et al., 2020), we speculated that V-ATPase may affect *Arabidopsis* FG development *via* auxin-mediated functions.

### Vacuolar H^+^-ATPase regulates nuclear spacing between the egg and the central cells through PIN-FORMED 1-mediated auxin transport during female gametophyte development

**FIGURE 6:**
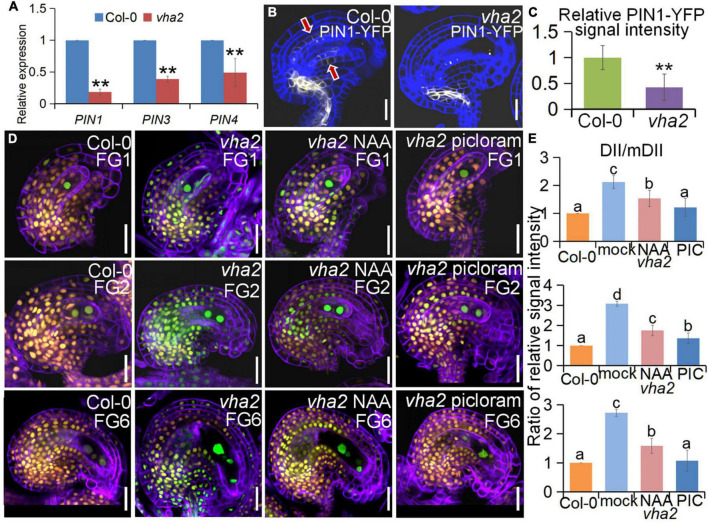
Lack of V-ATPase affects polar auxin transport and auxin distribution. **(A)** Relative transcript levels of *PIN1, PIN3*, and *PIN4* in inflorescence apices of Col-0 and *vha2.* Bars correspond to arithmetic means ± SE of three technical replicates of three biological replicates. Asterisks indicate significant difference (Studert’s tw0-tailed *t*-test: **P < 0.01). **(B)** PIN1-YFP express ion in Col-0 and *vha2* ovules. Bars = 20 μm. Graphs represent typical phenotype of all samples. **(C)** Quantitative analysis of PIN1-YFP fluorescence level in ovules. Bars correspond to relative YFP signal intensity. Relative fluorescence signal data were extracted from normalized mean gray levels in the above lines. Values correspond to arithmetic means ± SE of three biological replicates (*n* = 20). Asterisks indicate significant difference (Student’s two-tailed *t*-test: **P < 0.01). **(D)** R2D2 expression in Col-0 and *vha2* ovules under picloram and NAA treatment during FG development. (orange for mDII-tdTomato and green for DII-Venus). Cell walls were stained with FB28 (purple). Bars = 20 μm. Graphs represent the typical phenotype of all samples. **(E)** Quantification of fluorescent signals of R2D2 (DII/mDII). Relative fluorescence signal data were extracted from normalized mean gray levels in the above lines. Values correspond to arithmetic means ± SE of three biological replicates (*n* = 20). Lowercase letters indicate statistically significant differences between different stages (*P* < 0.05). 10 ovules shown in the statistical graph come from 3 different inflorescence apices.

At the late stages of FG development, auxin concentration in the embryo sac is relatively low and auxin transport in the sporophyte region surrounding the embryo sac mainly relies on the PIN1 protein (Luca et al., 2013; Panoli et al., 2015). Since our previous studies found that tonoplast proton pumps activities affect auxin gradient by altering the expression level and polar localization of PIN1 during embryonic development (Jiang et al., 2020), we first quantified the expression level of *PIN1* gene as well as other genes associated with auxin biosynthesis and response in FGs, using qRT-PCR. We found that the expression level of *PIN* gene family members was generally lower in *vha2* inflorescence (Figure 6A), especially that of *PIN1*, so was the expression of genes associated with auxin biosynthesis and signaling (Supplementary Figure 6). This result indicated that the auxin concentrations in *vha2* inflorescence is generally low. We then detected fluorescent signals of PIN1-YFP in Col-0 and *vha2* ovules and found that, PIN1-YFP signal in *vha2* FG4 embryo sacs was indeed significantly weaker than that in Col-0 (Figures 6B,C). This reduced PIN1-YFP signal in *vha2* FG4 embryo sacs indicated that the transportation of auxin in ovules is impeded, and that the inferior auxin content in the *vha2* ovules might be a signal to affect the movement of the central cell to the proper position and increase the distance between the central cell and the egg cell.

**FIGURE 7:**
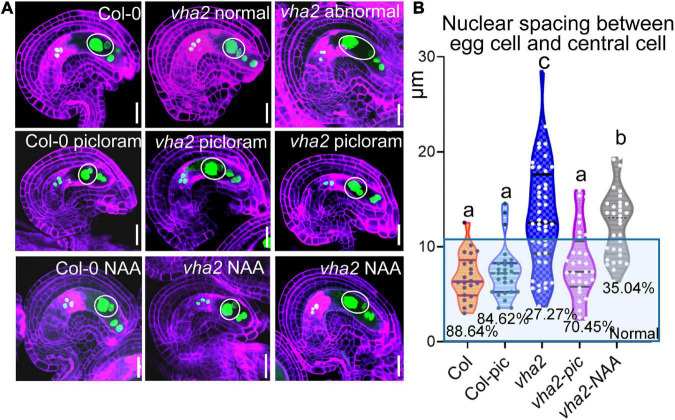
Picloram rescues the enlarged nuclear spacing of egg cells and central cells in *vha2* through PIN-independent pathway. **(A)** Nuclear spacing of Col-0 and *vha2* under picloram and NAA treatment at FG6 stage visualized using *ProES1:H2B-GFP* (green) by fluorescence microscopy. Cell walls were stained with FB28 (purple). Inflorescence apices grew for 2 days following 1 μM picloram or 1 μM NAA treatment for 24 h. In the white circles are nuclear of egg cells and central cells. Graphs represent the typical phenotype of all samples. Bars = 20 μm. **(B)** Statistics analysis of nuclear spacing between egg cell and central cell in Col and *vha2.* Dots in the blue box represent normal samples with nuclear spacing of egg cells and central cells less than 10 μm, and the proportions are shown in the chart. Lowercase letters indicate statistically significant differences between different stages (*P* < 0.05). 40 ovules shown in the statistical graph come from 10 different inflorescence apices.

To further validate our results, we treated Col-0 and *vha2* inflorescences *in vitro* with auxin analogs, picloram and NAA, respectively, and then examined the content of auxin using an auxin-level marker R2D2 (a semiquantitative and rapid auxin-input reporter with DII-Venus and mDII-tdTomato) after treatment, in which the absence of DII fluorescence marks auxin accumulation (Benková et al., 2003; Liao et al., 2015). Transport of picloram need the plasma membrane-bound carrier PIC30, rather than PIN family proteins that NAA transportations relies on. We found that the overall auxin level of *vha2* ovules along development is lower than that of Col-0 (Figures 6D,E). Auxin levels in *vha2* ovule were slightly and greatly restored by NAA or picloram treatment, respectively (Figures 6D,E). Notably, picloram treatment restored nuclear spacing in *vha2* embryo sacs to levels indistinguishable from Col-0 (Figures 7A,B), while NAA treatment hardly rescued the abnormal nuclear spacing albeit that a slight increase in normal proportions was observed (Figures 7A,B). These results indicated that, in *vha2*, PIN1 alone is not enough to transport sufficient NAA into ovules, resulting in the enlarged nuclear spacing. On the contrary, transportation of picloram does not require the participation of PIN1, so picloram could reach the ovules and to restore the abnormal nuclear distance between the egg cell and central cell.

**FIGURE 8:**
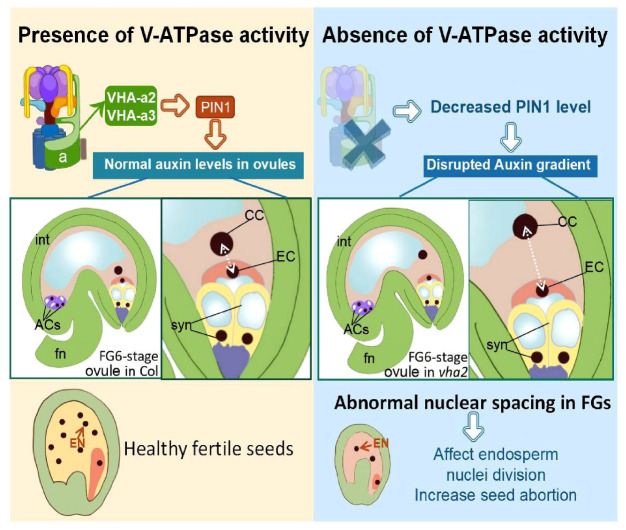
Schematic diagram of the role of V-ATPase on FGs and later endosperm development. Fn, funiculus; syn, synergid cell; EC, egg cell; CC, central cell; ACs, antipodal cells; int, integuments (inner and outer).

In conclusion, V-ATPase regulates auxin levels in ovules through coordinating the content and localization of PIN-FORMED 1 (PIN1) protein, hence influencing nuclear spacing between centra cell and egg cell, and subsequent endosperm development (Figure 8).

## Discussion

The life history of higher plants features an alternation between diploid sporophytes and haploid gametophytes. As model organism of angiosperms, *Arabidopsis* has been widely used to elucidate the underlying mechanisms of gametophyte developmental. In this study, we demonstrated that V-ATPase is essential for male and FG development, stigma patterning and early endosperm cell division. In this process, PIN1-mediated auxin transport is involved in the regulation of nuclear position downstream of the V-ATPase activity during FG development. These findings extend our understanding of the function of the tonoplast proton pump and auxin in the reproductive development of seed plants.

### Vacuolar H^+^-ATPase has multiple regulatory functions in male and female gametophytes and seed development

In this study, the triple mutant *fap3* lacking both tonoplast proton pumps was first used as the research material. Nevertheless, its severely impaired growth and uncompleted fertilizations in more than 90% gametophytes (Supplementary Figure 1) demonstrated that the lack of both tonoplast proton pumps severely affects the development of male and FGs, and that *fap3* is not suited to study the functions of the two tonoplast proton pumps during gametophyte development at the same time. Therefore, we decided to study two tonoplast proton pumps separately. Since the growth status, together with male and female transmission rate of *fugu5-1* mutant was comparable to that of Col-0, our work started to focus on V-ATPase instead of V-PPase.

After mutations of the a2 and a3 subunits that are responsible for tonoplast targeting of V-ATPase, male gametophytes in 26% (12/46) *Arabidopsis* anthers were arrested in PMI phase with large vacuoles (Figure 3). PMI is accompanied by vacuolar dynamics changes: a large central vacuole can be converted into several smaller vacuoles by convolution or fission (Yoko et al., 2003). It is well-known that endomembrane system proteins, including V-ATPases, are critical for the development of PMI (Whitley et al., 2009; Dettmer et al., 2010; Feng et al., 2017; Zhang et al., 2018). Our previous studies found that V-ATPase regulates the morphology and distribution of vacuoles during embryonic and seedling development, and that the loss of V-ATPase function in *vha2* male gametophytes results in the failure of 26% (12/46) large central vacuoles to transform into small ones.

During flower development of *vha2*, ovules in 17% (19/112) abnormal flowers could develop up to the FG7 stage but could not complete fertilization, because the lack of an active V-ATPase severely affected stigma morphology. The growth of pollen tube was seriously hindered, as most pollen tubes did not grow to the micropylar end to fertilize the ovule (Supplementary Figure 3). Consequently, *vha2* finally produced less than ten seeds. From FG development to double fertilization, the egg cell, as a female gamete, fuses with one sperm cell to give rise to the embryo. Meanwhile, the central cell, as the largest cell for nutrient storage, fuses with another sperm cell to form the endosperm. In *vha2* normal flowers, loss of V-ATPase resulted in a mis-localization of the central cell, and the subsequent division speed of endosperm cell was affected as well (Figures 4, 5). This data indicates that the correct positioning of the nuclei of FG is essential for the double fertilization, and that V-ATPase plays a pivotal role in regulating the position of the nuclei.

### The phytohormone auxin is involved in the regulation of vacuolar H^+^-ATPase in the coordinated development of plant sporophyte and gametophyte

During ovule development, the spatiotemporal distribution of auxin is essential for coordinated development of sporophyte and gametophyte (Pagnussat et al., 2007; Bencivenga et al., 2011). However, mechanisms underlying the spatial and temporal distribution of auxin remain ill-known. Auxin is a long-distance transported phytohormone, and the gradient around FG relies on the polar auxin transporter PIN1 (Luca et al., 2013; Panoli et al., 2015). The intracellular localization of PIN1 depends on the coordination of the endomembrane trafficking system (Gälweiler et al., 1998; Steinmann et al., 1999; Huang et al., 2010; Dhonukshe et al., 2015). In this study, we demonstrated that tonoplast V-ATPase plays a crucial role in the fine regulation of auxin and its function in FG development in *Arabidopsis*.

The spatial and temporal distribution of auxin in ovules is under the tight control of PIN1 (Luca et al., 2013; Panoli et al., 2015). Long-term exposure to NPA or BFA treatment disrupts FG development, a phenotype that has been reported to occur in *pin1* mutants (Wang et al., 2021). In line with previous findings (Luca et al., 2013; Panoli et al., 2015), observation of the R2D2 auxin marker in *Arabidopsis* ovules confirmed that DII expression in FG is higher than that in the sporophyte (Figure 6), which implies that the auxin content in *Arabidopsis* FG is relatively low. Loss of V-ATPase decreased auxin content in ovules throughout the FG developmental stages (Figure 6), and this was mainly attributed to the abnormal localization of PIN1 (Figure 6). This result was also supported by the fact that picloram, but not NAA, was able to restore the position of the central cell nuclei (Figure 7).

Eukaryotic V-ATPase has gained a lot of unconventional cellular functions during evolution, such as the regulation of important intracellular proteins (Cross and Muller, 2004; Marshansky and Futai, 2008). In the current work, the epistatic regulation of V-ATPase exerted on PIN1-mediated auxin transport in ovules during FG development has been illustrated. Collectively, our findings revealed a crucial role of V-ATPase in auxin-related FG development in *Arabidopsis* and enhanced our understanding of the functions of tonoplast proton pumps in this vital process.

## Data availability statement

The original contributions presented in this study are included in the article/Supplementary material, further inquiries can be directed to the corresponding author.

## Author contributions

W-HL designed the study, supervised the project, analyzed the data, organized the results, modified the manuscript, and acquired the funding. Y-TJ and J-XZ performed the experiment, analyzed the data, organized the results, and wrote the manuscript. R-HL and Y-CW helped performed the experiments and conducted association analysis. JS helped modify the manuscript. AF offered the materials and helped organized the results and the manuscript. All authors agreed to be accountable for the content of this manuscript, reviewed, and approved of the final manuscript.

## Footnotes

1
http://rsbweb.nih.gov/ij/download.html

## Abbreviations

DAG: days after seed germination
HAP: Hours after pollination
V-PPase: H**^+^**-translocating pyrophosphatase
V-ATPase: vacuolar H^+^-ATPase
PIC: picloram
MMC: megaspore mother cell
FM: functional megaspore.
