# Corrigendum: Tonoplast proton pumps regulate nuclear spacing of female gametophytes *via* mediating polar auxin transport in arabidopsis

**DOI:** 10.3389/fpls.2023.1152598

**Published:** 2023-02-10

**Authors:** Yu-Tong Jiang, Ji-Xuan Zheng, Rong-Han Li, Yu-Chen Wang, Jianxin Shi, Ali Ferjani, Wen-Hui Lin

**Affiliations:** ^1^ Laboratory of Metabolic and Developmental Sciences, School of Life Sciences and Biotechnology, The Joint International Research, Shanghai Jiao Tong University, Shanghai, China; ^2^ Zhiyuan College, Shanghai Jiao Tong University, Shanghai, China; ^3^ Department of Biology, Tokyo Gakugei University, Koganei, Japan; ^4^ Shanghai Collaborative Innovation Center of Agri-Seeds/Joint Center for Single Cell Biology, Shanghai Jiao Tong University, Shanghai, China

**Keywords:** plant vacuole, V-ATPase, female gametophyte, egg cell, central cell, endosperm

In the published article, there was a writing mistake in the schematic diagram of **Figure 8**. The cell abbreviations “EC” and “CC” were marked in the wrong place. The corrected **Figure 8** and its caption appear below:

**Figure 8:**
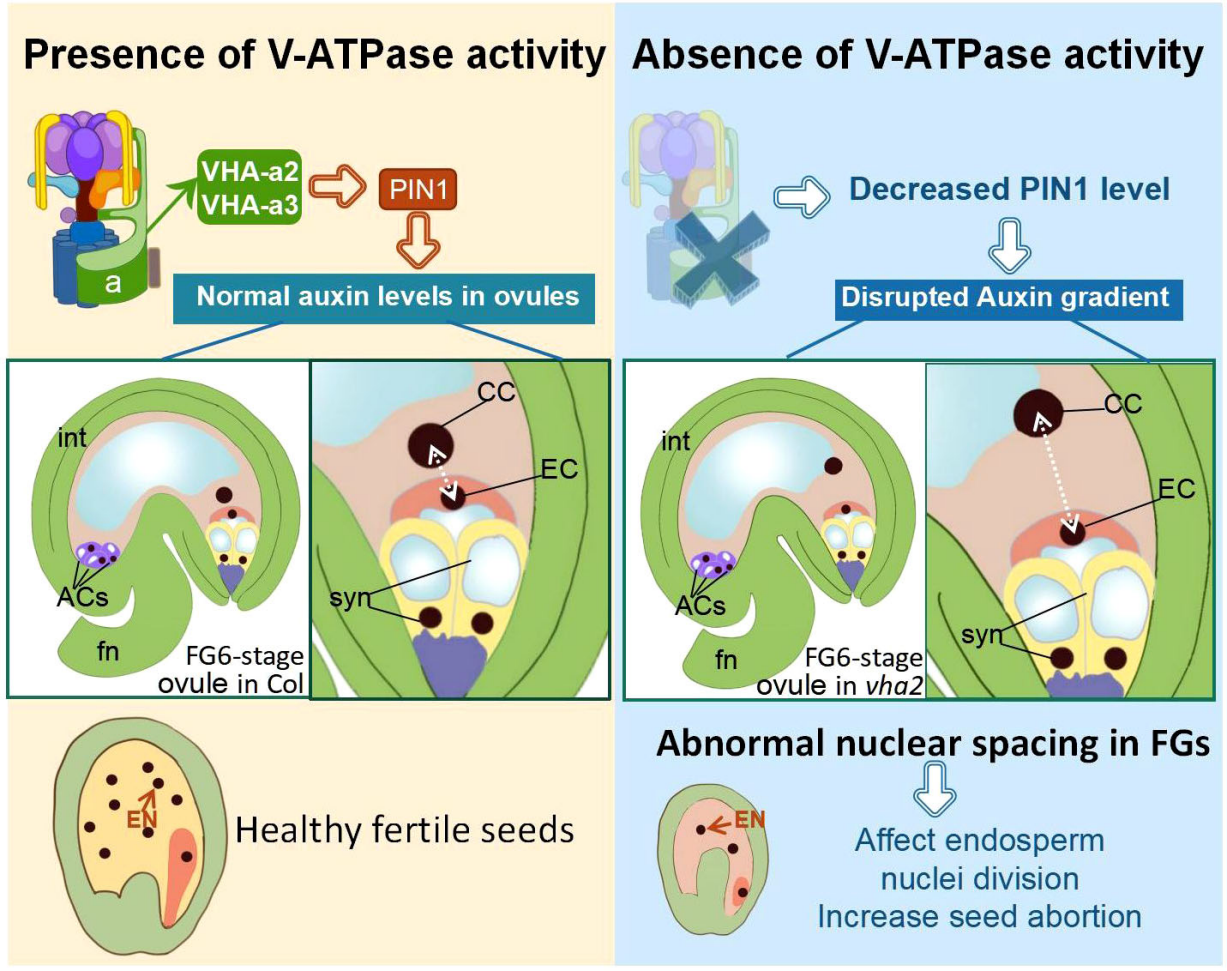
Schematic diagram of the role of V-ATPase on FGs and later endosperm development. fn funiculus, syn synergid cell, EC egg cell, CC central cell, ACs antipodal cells, int integuments (inner and outer).

The authors apologize for this error and state that this does not change the scientific conclusions of the article in any way. The original article has been updated.

