# Supplementary material for: Tonoplast proton pumps regulate nuclear spacing of female gametophytes via mediating polar auxin transport in Arabidopsis
Source: Front Plant Sci. 2022 Sep 13;13:1006735. doi: 10.3389/fpls.2022.1006735 (PMC9513470; doi:10.3389/fpls.2022.1006735)
Supplement: Supplementary file 1 [file Data_Sheet_1.pdf]

Table S1. Both male and female transmission of *FUGU5-1*, *VHA-a2*, *VHA-a3* are comparable to that of WT.

Parents	Genotype of Progenies				
	WT	*fugu5-1/+*	*fugu5-1*	Observed Ratio	Expected Ratio
♀ *fugu5-1/+* × ♂ WT	94	89	0	1:0.95[a]	1:1
♀ WT × ♂ *fugu5-1/+*	82	88	0	1:1.07[a]	1:1
♀ *fugu5-1/+* × ♂ *fugu5-1/+*	41	71	37	1:1.73:0.9[b]	1:2:1

Parents	Genotype of Progenies				
	WT	*vha-a2/+*	*vha-a2*	Observed Ratio	Expected Ratio
♀ *vha-a2/+* × ♂ WT	77	72	0	1:0.94[a]	1:1
♀ WT × ♂ *vha-a2/+*	92	87	0	1:0.95[a]	1:1
♀ *vha-a2/+* × ♂ *vha-a2/+*	29	59	24	1:2.03:0.89[b]	1:2:1

Parents	Genotype of Progenies				
	WT	*vha-a3/+*	*vha-a3*	Observed Ratio	Expected Ratio
♀ *vha-a3/+* × ♂ WT	73	81	0	1:1.11[a]	1:1
♀ WT × ♂ *vha-a3/+*	84	79	0	1:0.94[a]	1:1
♀ *vha-a3/+* × ♂ *vha-a3/+*	47	89	43	1:1.89:0.91[b]	1:2:1

a Not significantly different from the expected segregation ratio 1:1 ($\chi 2$, $P > 0.05$).

b Not significantly different from the expected segregation ratio 1:2:1 ($\chi 2$, $P > 0.05$).

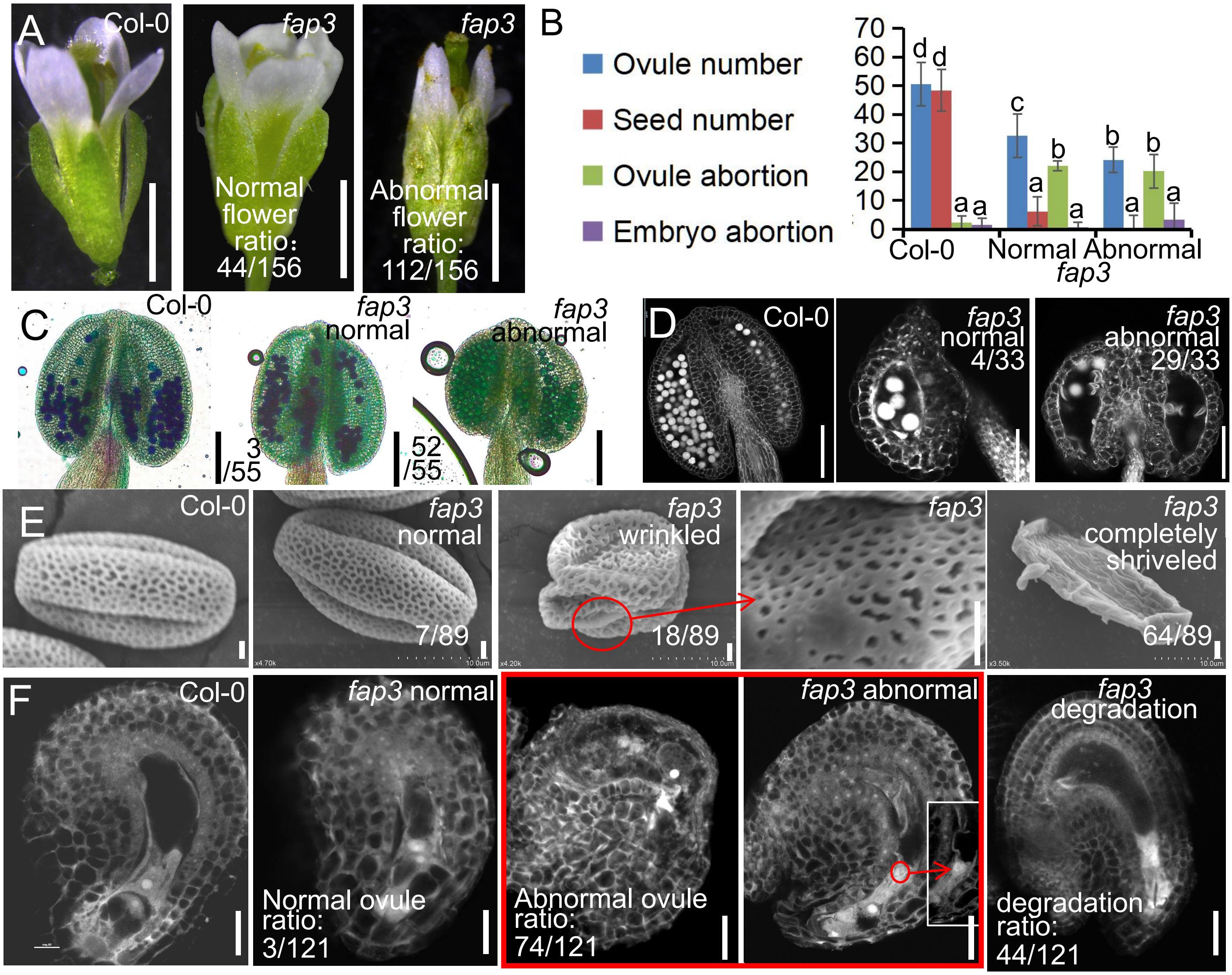

Figure S1 Lack of V-PPase and V-ATPase result in the defective development of pollen grains and female gametophytes.

(A) Flower development at stage-13 in Col-0 and *fap3*. Statistics on the ratio of normal and abnormal microspores in *fap3* are shown in each image. Bars = 1 mm.
(B) Statistics analysis of ovule number, seed number, ovule abortion, and embryo abortion in Col-0 and *fap3.* Bars represent the mean ± SD of three biological replicates (n =30). Lowercase letters indicate statistically significant differences between different stages (P<0.05). Theoretically, ovule number = seed number + ovule abortion + embryo abortion.
(C) Alexander's staining of pollen from Col-0 and *fap3*. The purplish red color indicates viable pollen. Statistics on the ratio of normal and abnormal anthers in *fap3* are shown in the bottom left corner of each image. Bars = 100 μm.
(D) Anthers visualized by fluorescence microscopy. Col-0 and *fap3* anthers at stage 12. Statistics on the ratio of normal and abnormal flowers in *fap3* are shown in each image. Bars = 100 μm.
(E) Scanning electron micrographs (SEMs) observation on pollen grains from Col-0 and *fap3.* Bars = 2 μm.
(F) Embryo sac development of Col-0 and *fap3* at late-FG6 stage visualized by fluorescence microscopy. FG cells are degraded with blurred bright areas; and abnormal FG is only abnormal in the nuclear location and overall morphology, but maintains the intact structural morphology of the cell. Two images circled in red box represent the abnormal FG phenotypes contained in *fap3*. Bars = 20 μm.

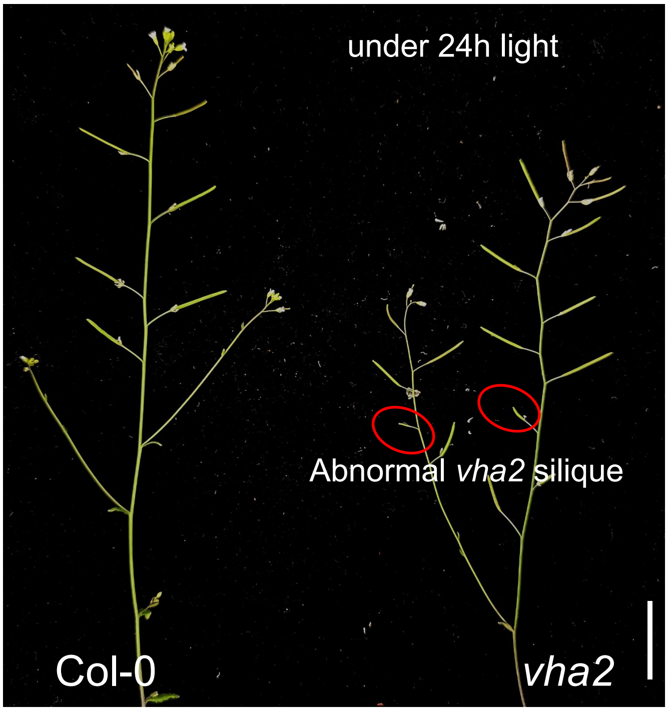

Figure S2. Reproductive growth of *vha2* compared with Col-0 under 24-hour-light culture. The red circles indicate siliques with a lower seed setting rate. Bar = 2 cm.

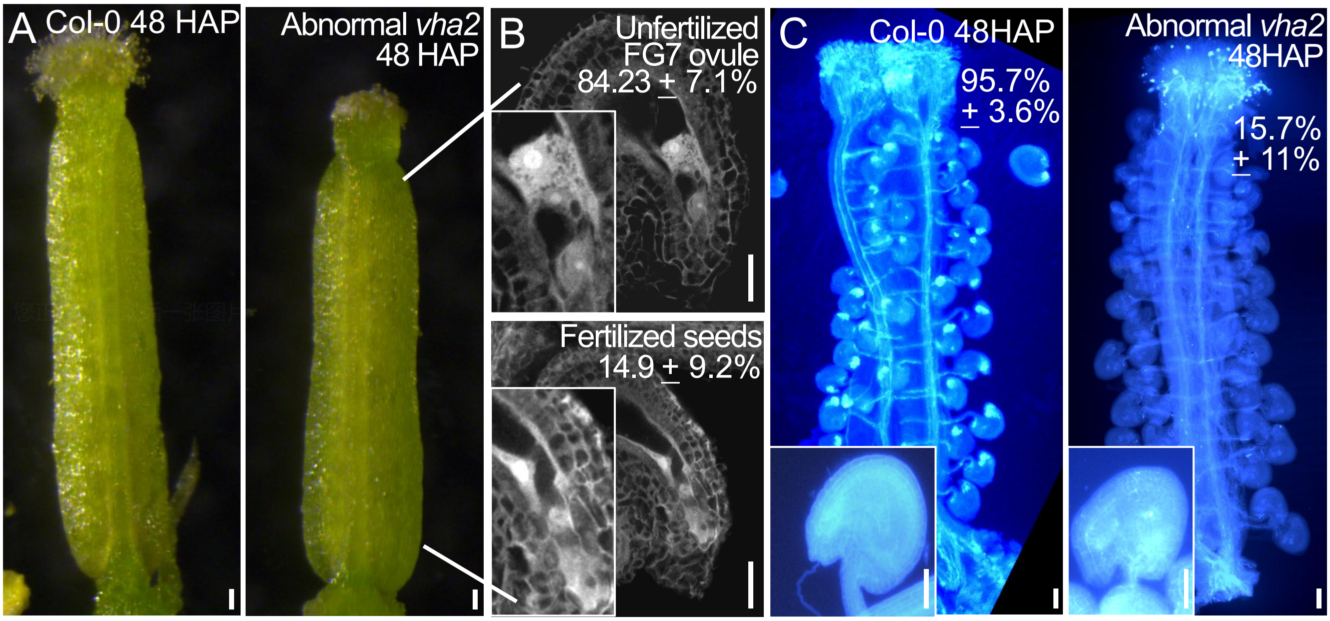

Figure S3. Abnormal *vha2* with abnormal stigma renders most ovules infertile.
(A) Stigma development at 48 HAP in Col-0 and abnormal *vha2*. Bars = 20 μm.
(B) Unfertilized ovule and fertilized seeds in 48-HAP siliques of abnormal *vha2*. Statistics on the ratio are shown in each image.
(C) Aniline blue staining of Col-0 and abnormal *vha2* pistils at 48 h after pollination (HAP) with Col-0 pollen. The whole pistil was overlaid by two overlapping images with Photoshop (Adobe). Numbers in (C) are quantification of targeted ovules out of total ovules. Results are means ± SD (n = 15). Bars = 20 μm.

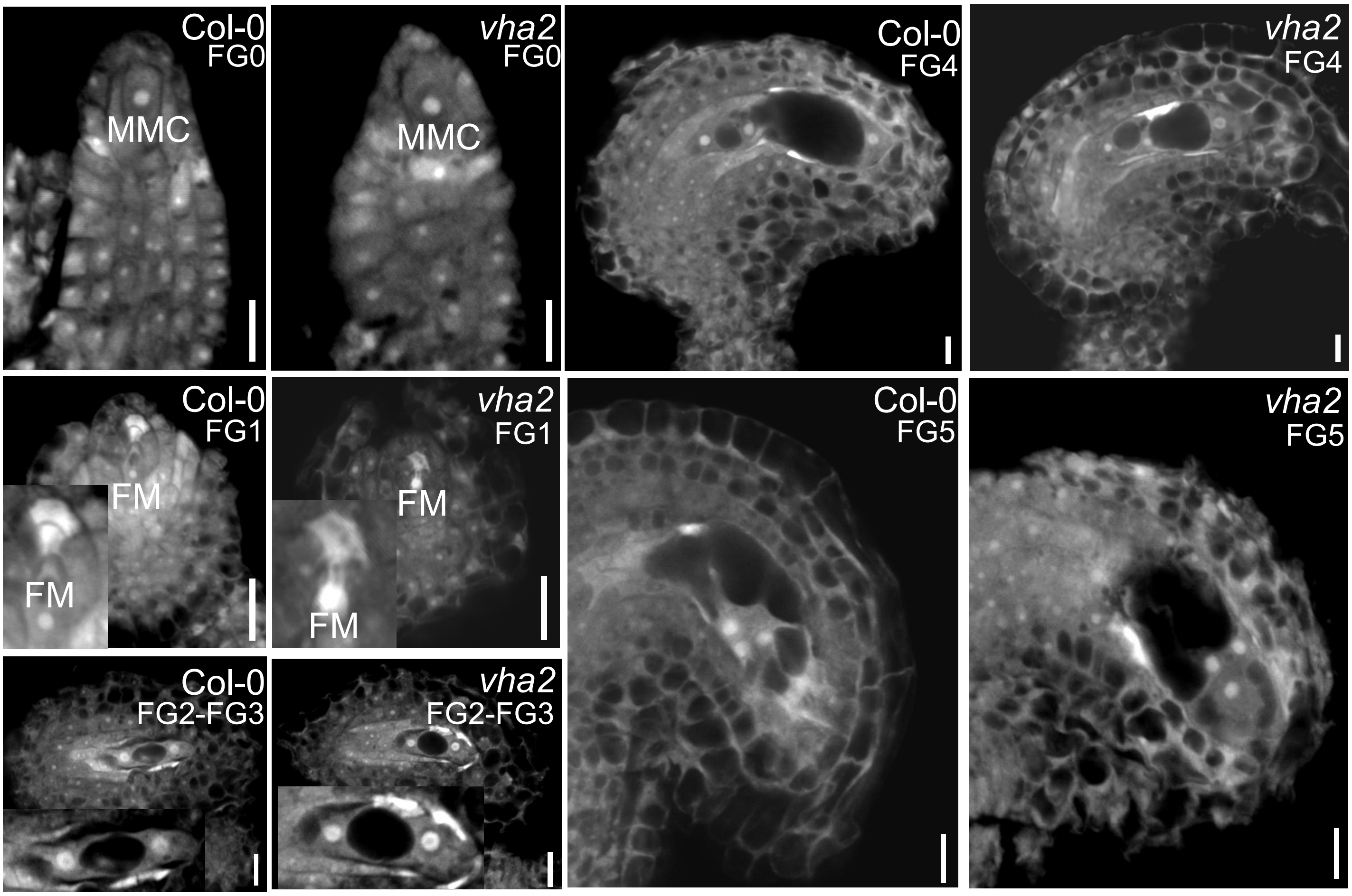

Figure S4 FG development is normal in *vha2*.
FG1-FG5-stage embryo sac development of Col-0 and *vha2* are visualized by fluorescence microscopy. Graphs represent typical phenotype of all samples (n=50). Bars = 10 μm.
MMC megaspore mother cell, FM functional megaspore.

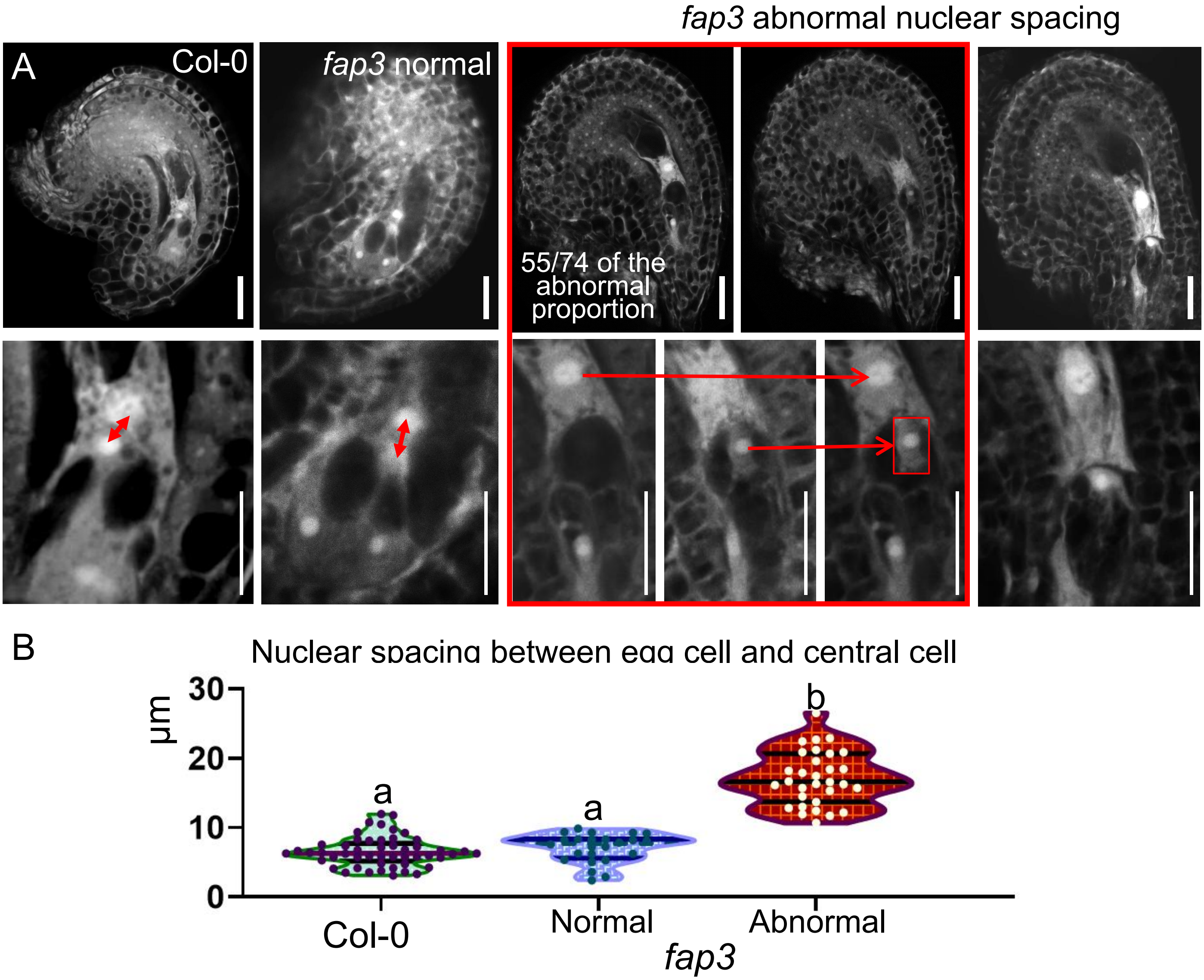

Figure S5 Nuclear-spacing of egg cells and central cells in *fap3.*
(A) Embryo sac development of Col-0 and *fap3* at late-FG6 to FG7 stage visualized by fluorescence microscopy. The picture below is an enlarged view of the nuclear spacing between the egg cell and central cell in the above image. In the red frame, the same ovule is shown at different angles. The red arrows point out the nuclear-spacing of egg cells and central cells. Graphs represent the typical phenotype of all samples. Bars = 20 μm. (B) Statistics analysis of nuclear spacing between egg cell and central cell in Col-0 and *fap3.* Below the red line is defined as normal spacing if the nuclear spacing between egg cell and central cell is less than 10 μm, and it was considered abnormal spacing if spacing exceeds 10 μm. Lowercase letters indicate statistically significant differences between different stages (P<0.05). 50 ovules shown in the statistical graph come from 10 different inflorescence apices.

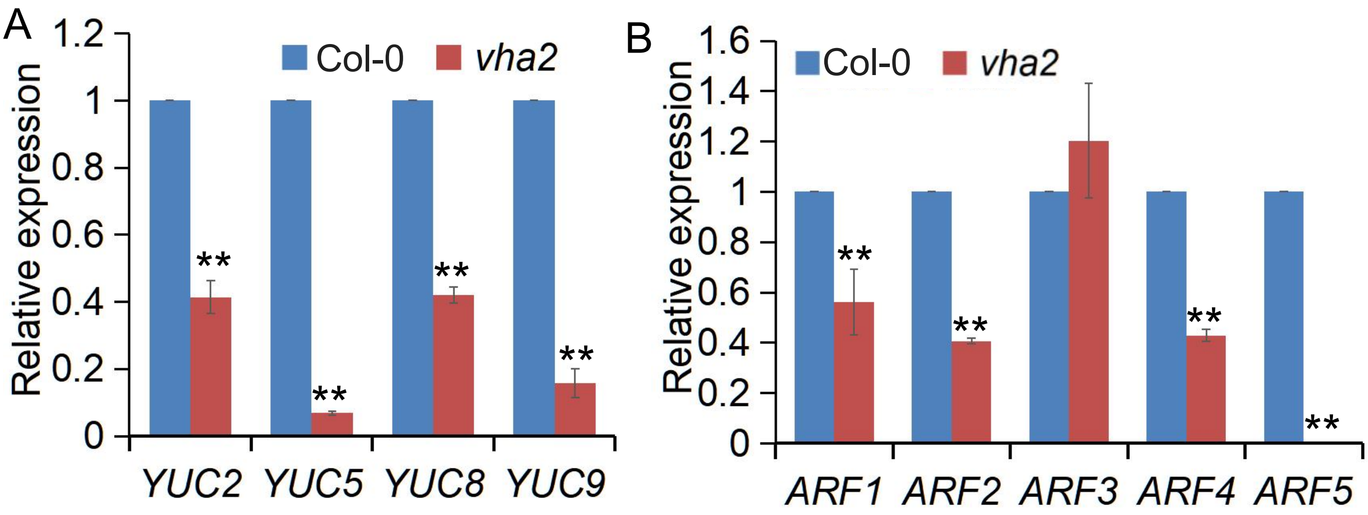

Figure S6 Relative transcript levels of *YUCCAs* (A) and *ARFs* (B) in inflorescence apices of Col-0 and *vha2.* Bars correspond to arithmetic means ± SE of three technical replicates of three biological replicates. Asterisks indicate significant difference (Student’s two-tailed t-test: **, $P < 0.01$).

Table S2. Primers used in this study	
Cloning	
VHA-a3-F	tctgattaacaggaTTTGATAGCTACTCTCTCC
VHA-a3-R	catactagttctGGATCCCTCGTCTTCGTTTGCCGTGA
qRT-PCR	
CPD-RT-F	CATGGAAGAAGCCAAAAAGATAACG
CPD-RT-R	CTTTGCGGTAAGTGGTGGAGA
DWF4-RT-F	CATGTCTCCAAGTATGGTAAGATAT
DWF4-RT-R	ATTTCCCAAGAATCCCACCTATACT
ACTIN-RT-F	CCGGTATTGTGCTCGATTCTG
ACTIN-RT-R	TTCCCGTTCTGCGGTAGTGG
PIN1-RT-F	TCTTCTCAAAGGCATGTATGGT
PIN1-RT-R	CGAAACAATAGATCCTGCTGTG
PIN3-RT-F	GGATTACGTGGTGATTTACTGC
PIN3-RT-R	GATGAACATTGTACTCCTTCGC
PIN4-RT-F	CGGATTTGTACTCCGTTCAATC
PIN4-RT-R	GTTTAGTTGAAACACCCGTACC
YUC2-RT-F	CAAGATCAAATGCGGAAAGACT
YUC2-RT-R	CCGAATAATGCATTACCCGTTT
YUC5-RT-F	GTTCTAGACATCGGAGCTATGG
YUC5-RT-R	GTGACTACGAGAGAACCGTTTA
YUC8-RT-F	AAATACGGTTTGAAACGACCAG
YUC8-RT-R	TCGACTTTGTTTCCGTTAAACC
YUC9-RT-F	CTCGTAGATGGTCAGAAGCTAG
YUC9-RT-R	AATGTCTTGAGCGATGTTAACG
ARF1-RT-F	GAGACTGTGGAGTACAAGAACA
ARF1-RT-R	CTCAGCTGCATTCATAGCATTT
ARF2-RT-F	GGATTTTCTGTTCTTAGGCGAC
ARF2-RT-R	ATATGTCTGAATCGCCACTCAT
ARF3-RT-F	TGTTGTTGACTCGAGTGATACA
ARF3-RT-R	TTTGCAAATATCAGAACCACCG
ARF4-RT-F	TGTACAAAGGTTCACAAGCAAG
ARF4-RT-R	AGTGTATCTTCCACACCACATT
ARF5-RT-F	GATAATTCTGGAGGCACTGGTA
ARF5-RT-R	CAAACATGCATTCGATAGCAGA
